# Quality of Care of Patients with Diabetes in Primary Health Services in Southeast Brazil

**DOI:** 10.1155/2017/1709807

**Published:** 2017-10-10

**Authors:** Christiane Chaves Augusto Leite Simão, Mônica Barros Costa, Fernando Antônio Basile Colugnati, Elaine Amaral de Paula, Chislene Pereira Vanelli, Rogério Baumgratz de Paula

**Affiliations:** Postgraduate Program in Brazilian Health, School of Medicine, Federal University of Juiz de Fora, Avenida Eugênio do Nascimento, s/n, Bairro Dom Bosco, 36.038-330 Juiz de Fora, MG, Brazil

## Abstract

**Background:**

Diabetes management involves multiple aspects that go beyond drug therapy as a way of providing high quality care. The objective of this study was to describe quality of care indicators for individuals with diabetes in southeast Brazil and to explore associations among these indicators.

**Methods:**

In this cross-sectional, observational study, health care providers filled out a questionnaire addressing health care structure and processes at 14 primary health care units (PHCUs). Clinical and laboratory data of diabetic patients attending the PHCUs and from patients referred to a secondary health care (SHC) center were collected.

**Results:**

There was a shortage of professionals in 53.8% of the PHCUs besides a high proportion of problems regarding referrals to SHC. At the PHCU, glycated hemoglobin results were available only in half of the medical records. A low rate of adequate glycemic control was also observed. An association between structure and process indicators and the outcomes analyzed was not found.

**Conclusion:**

Major deficiencies were found in the structure and processes of the PHCUs, in addition to unsatisfactory diabetes care outcomes. However, no association between structure, process, and outcomes was found.

## 1. Introduction

The phenomenon of epidemiological transition, characterized by a change of focus from acute to chronic health conditions (CHC) as causes of morbidity and mortality, is a challenge to public health worldwide [1] as well as in Brazil, where the health needs have changed, requiring continuous and proactive attention focused on permanent care [2].

As a consequence, Mendes adapted the Chronic Care Model to the Brazilian Unified Health Care System [2]. In this proposal, health services are interconnected with the main purpose of delivering comprehensive care for specific health conditions [2], constituting the Health Care Networks (HCN). In this model of chronic care, the risk stratification of a specific population is a fundamental task of primary health care (PHC), which has a managerial role in the HCN [2].

It is noteworthy that essential elements of primary care include accessibility, longitudinality, comprehensiveness, and coordination [3]. In Brazil, PHC is provided through the Family Health Strategy (FHS), a successful program created in 1994. Since then, health indicators have improved in the country [4–7]. However, disparities in health care, particularly related to funding and organization, have been reported [6]. Restraints for an effective HCN coordination include lack of knowledge by PHC professionals regarding patient referral and difficulties in patients' access to specialized care [8].

As a CHC, diabetes is becoming more prevalent, affecting approximately 7% of the adult Brazilian population [9] and most individuals with diabetes still demonstrate poor glycemic control [10, 11]. It is worth noting that the morbidity and mortality associated with diabetes are related mainly to chronic complications of the disease that can be prevented by optimal metabolic control, along with self-care measures, periodic clinical follow-up, and prompt therapeutic intervention. Thus, a proper understanding of the clinical characteristics of the patients and the organizational aspects of the health system may help to improve decisions regarding the care of patients with diabetes and other CHCs [12].

The objective of this study was to describe the structure and process of PHC units (PHCUs) and the outcomes of patients with diabetes in a medium-sized municipality in southeast Brazil and to explore the association between these indicators.

## 2. Methods

In this observational cross-sectional study, PHC was evaluated in a medium-sized municipality in southeastern Brazil. Elements of structure, process, and results of the assistance were evaluated, according to the triad proposed by Donabedian [13].

Fourteen PHCUs were selected based on the electronic records of patients receiving shared attention at a SHC service. The units that had made the largest and the smallest number of referrals to the SHCU in each of the seven health regions of the city were included.

In order to analyze PHC structure and process an adapted questionnaire [14] and PHCU medical records were used. To evaluate the outcomes, data from charts in PHCU and from the SHC medical records were utilized. Data from the SHC unit were analyzed as a PHC outcome, as they were collected exclusively from each patient's first consultation at the specialized care unit.

Intending to analyze the physical and organizational characteristics of the PHCUs, a semistructured questionnaire, adapted from Pereira [14], was presented to the manager, one physician, and one community health agent (CHA) at each of the selected PHCUs, after obtaining informed consent.

The questionnaire included items evaluating the services provided to patients with CHCs, which was divided, a posteriori, according to the categorization proposed by Donabedian [13] to evaluate structure and processes of the health care. Structure-related questions assessed material and human resources, as well as organizational structure. Process-related questions analyzed strategies to provide medical care involving issues concerning the health team. The questions had dichotomous responses and were grouped in order to compose indicators summarizing the structure and process at each PHCU. Each response which indicated compliance with recommended guidelines [15–17] received one point, and the sum of these points was used to classify the structure and processes in each unit.

In each PHCU, the medical records of the patients diagnosed with diabetes, aged 18 years or more and of both sexes, who had had at least one medical appointment since 2010, were analyzed. The last two measurements of blood pressure (BP), fasting plasma glucose (FPG), glycated hemoglobin (HbA1c), and serum creatinine, along with estimated glomerular filtration rates (eGFR) calculated using the MDRD equation, were retrieved.

Data obtained from the PHCUs medical records were used as both indicators of process and outcomes, fulfilling the evaluation triad proposed by Donabedian [13]. As process indicators, the glycemic monitoring rate, defined as the proportion of individuals with diabetes with at least one HbA1c result recorded in the medical charts during the year that preceded the collection of the data, was calculated. In addition, serum creatinine was used to calculate the renal monitoring rate. The results of BP, FPG, HbA1c, and eGFR were analyzed as outcomes of care.

In order to evaluate the outcomes, the computerized medical records of the patients in the SHC outpatient clinic were also analyzed. Data from the SHC unit were analyzed as a PHC outcome, as they were collected when the patients were initially admitted to the specialized care unit between August 2010 and December 2014. At this SHC unit, the parameters age, sex, educational level, income, tobacco use, alcohol use, medications in use, practice of physical exercise, weight, height, body mass index (BMI), BP, HbA1c, LDL cholesterol, and eGFR were collected from the patients' initial records. Those medical records of patients younger than 18 years of age and those in whom the absence of data in the medical records would compromise the research were excluded.

In the data analysis, the target levels of ≤130 mg/dL for FPG, <7.0% for HbA1c, and <140 × 90 mmHg were considered as indicative of adequate control in individuals with diabetes [15–17]. An eGFR ≤60 mL/min/1,73 m^2^ on a single occasion was regarded as suggestive of chronic kidney disease [15–17]. Respecting the frequency in which the tests were carried out in the PHCU, intervals shorter than 6 months for BP, FPG, and HbA1c were considered adequate [15–17]. With regard to the measurement of creatinine and calculation of eGFR, intervals shorter than 12 months were seen as appropriate [15–17].

### 2.1. Statistical Analysis

Categorical and quantitative variables were analyzed descriptively. The results are presented in percentages, mean and standard deviation, and median and interquartile interval. Associations between elements of the PHCUs' structure and process and the patients' clinical parameters upon admission to the SHC unit were explored with linear regression with random effects, using the data from the PHCUs as a conglomerate as we assumed that data from a single PHCU were not completely independent. The data were analyzed with the software SPSS 21.0 (SPSS Inc., Chicago, USA).

## 3. Results

### 3.1. Analysis of Questionnaires

Fourteen physicians, 13 managers, and 13 CHAs were interviewed due to the fact that one of the selected PHCUs was not a FHS unit and lacked CHAs, and another had no manager when the data were collected. The average working years in PHC was 13 ± 6.7 among physicians, 14 ± 7.6 among managers, and 9 ± 3.0 among CHAs. The most important aspects of structure and processes of the PHCUs are shown in Table 1.

### 3.2. Analysis of Medical Records

#### 3.2.1. Primary Health Care Units

A total of 844 medical records of registered patients with diabetes were analyzed, of whom 248 were excluded for having the most recent data recorded before 2010. These data were not collected from two of the PHCUs, one that had no record of patients with diabetes and another that declined our request to review the medical records.

Among the 596 medical records evaluated, the mean age of the patients was 64 ± 12.8 years and 67.3% were female. In addition, a total of 501 (83.9%) patients had a diagnosis of hypertension in association with diabetes.

Regarding the information from physical examination and tests described in the medical records, a glycemic monitoring rate of 22.7% and a renal monitoring rate of 33.4% were observed. The mean eGFR was 75 ± 32.7 mL/min/1,73 m^2^, and 25.5% of the patients had an eGFR below 60 mL/min based on the last test result. Furthermore, BP was uncontrolled in 47.0% of the patients. The results of the remaining tests, along with their availability in the medical records and compliance with the recommended interval in the care of patients with diabetes, are shown in Table 2.

#### 3.2.2. Secondary Health Care Unit

A total of 2,717 medical records were evaluated initially, but 23 were excluded because patients were under 18 years of age or chart data were incomprehensible, yielding 2,695 analyzed charts. The mean age of the patients was 60 ± 13.1 years and the mean annual household income was US$7,533.36. An analysis of the results of tests which were considered to be important parameters of primary health care outcomes showed that 81.4% of the individuals admitted to the SHC unit were overweight or obese, 18.7% of them had HbA1c below 7%, 36.5% had LDL cholesterol below 100 mg/dL, and 64.4% of the patients had an eGFR above 60 mL/min/1,73 m^2^.  Table 3 shows the characteristics of these individuals, along with the subgroup of patients who were referred to by the 14 PHCUs whose structure and process were evaluated using the questionnaire.

A possible influence of characteristics related to the PHCUs' structure and processes on health care outcomes, inferred by the characteristics of the patients in their initial appointment at the SHC unit, are presented in Table 4. As shown in this table, no significant association was observed.

## 4. Discussion

Even though PHC is extremely important within the context of the HCNs, a high frequency of deficiencies in structure and, mainly, in processes in the PHCUs evaluated in this study were observed. Also, a high proportion of patients with diabetes did not reach the recommended targets for most health care outcomes.

The success of HCNs as a strategy to improve health care depends on the effectiveness of the PHC in terms of resolution, accountability, and coordination [15]. These attributes were hindered in our sample by the shortage of material resources in the PHCUs' structure. As for human resources, a shortage of CHAs in more than half of the units evaluated was observed, a finding similar to a Brazilian Midwest study that found a lack of CHA in 59.5% of the teams evaluated [18]. It is worth noting that effective work by the CHAs is regarded as one of the most important components in FHS as it represents a link between the PHC and the community [6].

The high frequency of barriers in referring patients to SHC, reported in 70% of the PHCUs, must be emphasized, especially with respect to the site and criteria for the referral. Several studies have demonstrated this gap in coordination of care in the Brazilian Unified Health Care System [19, 20]. Training in health care protocols and referral process has been suggested as an effective strategy to improve quality indicators [21] and could be a solution for these shortcomings.

When the medical records at the PHCUs were analyzed, important process-related problems were also observed. Only 51.2% of the records of individuals with diabetes had results of HbA1c tests. Regarding the interval between these tests, the period of 6 months recommended by most guidelines for patients well controlled and with a stable disease [15, 16] was observed in only 14.4% of the patients. The glycemic monitoring rate of 22.7% in the PHCUs in our study was also below that described in the literature, which ranges from 30% to above 94% in developed countries [22–24].

Regarding chronic complications of diabetes, the renal monitoring rate was inadequate when compared with rates of 57.1–97.7% reported by Stone et al. in eight European countries evaluated [23]. Low frequency of ophthalmoscopy and of screening for loss of protective plantar sensation was described by the physicians in this study. These rates are lower than those reported previously in Brazil [25]. Actually, the frequency of both examinations varies worldwide [22, 23, 26].

In terms of health care results, a low percentage of patients with adequate metabolic control were found. Levels of HbA1c below 7% were observed in less than a fifth of the medical records. These rates are lower than those described in Brazilian studies [10, 11] and contrast even more with data from Europe and the United States, which range from 35.7% to 70.5% [23, 24]. However, the findings of our study should be interpreted with caution given the low frequency of HbA1c results available in the medical records analyzed.

With regard to comorbidities associated with diabetes, the outcomes were also unsatisfactory. Adequate BP control was observed in 53% of the patients with diabetes in the PHCUs, a rate higher than that described in the general Brazilian population (19.6%) [17], but lower than the data observed in an endocrinology outpatient clinic in south Brazil [27]. In other countries, most of studies found a higher percentage of adequate BP control in patients with diabetes [24, 26]. Excessive body weight was a frequent finding in the studied sample, and similar data was described in several studies related to the quality of diabetes care [11, 23, 27]. The proportion of patients with LDL cholesterol levels below 100 mg/dL in the present study was lower than that reported in patients assisted in another Brazilian SHC service [27]. Actually, the rates observed in different populations are very heterogeneous [22, 23].

With respect to the treatment of diabetes, the population admitted to the SHC unit in this study showed a lower frequency of insulin use when compared to countries such as Germany, Italy, Sweden, United Kingdom [23] and to Brazilian studies [10, 11]. However, the population analyzed in the present study included patients for whom insulin should have been prescribed and could not be implemented in a PHCU. In contrast, the frequency of use of aspirin and statin was greater than that described in other patients with diabetes [26].

No statistical or clinical significance was found when possible associations among parameters of structure and process with clinical outcomes were evaluated. Similar data have been described by Wesselink et al. in the Netherlands [28] and by Sidorenkov et al., in a systematic review, thus pointing out to the need for robust indicators of health care quality [29]. In addition, it is worth noting that the results are not direct measures of the initiatives of the health professionals and may be influenced by other factors, such as lifestyle and socioeconomic circumstances, over which the institutions and care providers have no control [30].

The questionnaire used to evaluate the PHCUs was an adapted version and could be pinpointed as a limitation in the present study. However, the evaluation of the quality of medical care has been challenging for decades [13], possibly due to the absence of a consolidated instrument for this purpose. On the other hand, it must be emphasized that this is a real-life study whose findings could contribute to health policy planning in primary care.

## 5. Conclusion

In the present study, major deficiencies were found in the structure and processes of the PHCUs, in addition to unsatisfactory diabetes care outcomes. However, no association between structure, processes, and outcomes was found. Additional studies are required to identify the most relevant indicators of structure and processes in order to improve the quality of care of individuals with diabetes.

## Figures and Tables

**Table 1:** Elements of structure and process of the 14 evaluated PHCUs according to the questionnaire.

*Structure*	
Material resources	
Availability of offices for consultation of patients with diabetes by a physician and a nurse on the same shift	38.5%
Adequate number of blood glucose test strips	69.2%
Sufficient number of diabetes medications to meet the demand of the unit	30.8%
Availability of educational material on diabetes for the population	69.2%
Human resources	
Completeness of the FHS team	46.2%
Most frequently lacking professional	CHA (53.8%)
Physicians and nurses trained in FHS	100%
Nursing assistants trained in FHS	72.7%
CHAs trained in FHS	81.8%
Organizational structure	
Existence of scheduled nursing appointments for diabetes care	61.5%
Existence of scheduled medical consultation for patients with diabetes	92.3%
Active retrievability of diabetic patients who missed scheduled appointments	46.2%
Problems related to coordination of care of patients with diabetes	69.2%
Uncertainty about the unit where the patient should be referred to	69.2%
Unawareness of the criteria for referral	69.2%
Shortage of available appointments	57.1%

*Processes*	
Recommendations about healthy eating and physical activity	100%
Guidance regarding insulin application when indicated	71.4%
Requests for FPG, HbA1c, creatinine, lipid profile, and urinalysis	100%
Adequate return of test exams	21.4%
BP measurement during all appointments	78.6%
Weight and height measurement during all appointments	50%
Screening for loss of protective plantar sensation in the feet	14.3%
Screening for retinopathy	7.1%

FHS: Family Health Strategy; CHA: community health agent; FPG: fasting plasma glucose; HbA1c: glycated hemoglobin.

**Table 2:** Clinical and laboratory data of patients with diabetes in the evaluated primary health care units.

Parameters	Availability of information, *n* (%)	Compliance with the recommended interval, *n* (%)	Mean ± SD	Frequency of adequate control, *n* (%)
SPB (mmHg)	596 (100%)	367 (61.6%)	134 ± 20.3	347 (58.2%)
DBP (mmHg)	596 (100%)	367 (61.6%)	82 ± 11.0	432 (72.5%)
FPG (mg/dL)	537 (90.1%)	138 (23.1%)	154 ± 69.0	274 (46.0%)
HbA1c (%)	305 (51.2%)	86 (14.4%)	8 ± 2.3	111 (18.6%)

SBP: systolic blood pressure; DBP: diastolic blood pressure; FPG: fasting plasma glucose; HbA1c: glycated hemoglobin. Recommended intervals: blood pressure ≤ 6 months; HbA1c ≤ 6 months; FPG ≤ 6 months.

**Table 3:** Characteristics of the patients with diabetes admitted to secondary health care outpatient clinics (*n* = 2,695) subdivided into those referred from the 14 PHCUs studied (*n* = 726) and those from other PHCUs (*n* = 1,969).

	Total	PHCUs studied	Other PHCUs	*p* value
*Sociodemographic data*				
Sex (% women)	61.9%	62%	61.9%	0.156
Race (% nonwhite)	55.3%	57.4%	54.5%	0.108
Education level				0.291
Illiterate	6.1%	4.7%	6.7%	
Incomplete elementary education	61.6%	64.2%	60.6%	
Complete elementary education	15.8%	15.7%	15.8%	
Complete secondary education	13.8%	12.7%	14.3%	
Complete higher education	1.9%	2.2%	1.8%	
*Clinical data*				
Sedentary lifestyle	21.7%	22.3%	21.5%	0.113
Alcohol abuse	13.7%	12.5%	14.1%	0.562
Current smoking	9.0%	9.4%	8.8%	0.433
BMI (mean, kg/m^2^)	31,00	31,04	30,91	0.670
Controlled BP	43.9%	42.8%	45.5%	0.202
Insulin use	21%	24.9%	19.5%	0.002
Metformin use	69%	68.2%	69.2%	0.622
Sulphonylurea use (%)	41%	37.3%	42.1%	0.027
ACEI/ARB use (%)	65%	68.7%	63.9%	0.020
Statin use (%)	55%	58.1%	53.4%	0.300
Aspirin use (%)	44%	47.4%	42.3%	0.018
*Laboratory data*				
HbA1c (mean, %)	9.3	9.21	9.31	0.457
LDL (mean, mg/dL)	116	116,71	116.17	0.848
eGFR (mean, mL/min)	78	75.69	78.35	0.127

BMI: body mass index; BP: blood pressure; ACEI: angiotensin-converting enzyme inhibitor; ARB: angiotensin receptor blocker; HbA1c: glycated hemoglobin; LDL: LDL cholesterol; eGFR: estimated glomerular filtration rate (MDRD).

**Table 4:** Associations between structure and processes at primary health care and outcomes at secondary health care.

Clinical parameters	Parameters used to evaluate the quality of health care	*β*	95% CI
HbA1c	Structure points	0.005	(−0.058; 0.068)
	Process points	0.236	(−0.063; 0.535)
	Proportion with HbA1c results in the previous year	0.399	(−2.740; 3.538)
	Proportion with creatinine results in the previous year	0.324	(−2.553; 3.201)

LDL cholesterol	Structure points	−1.903	(−3.269; −0.537)
	Process points	7.656	(0.492; 14.821)
	Proportion with HbA1c results in the previous year	−68.814	(−141.718; 4.090)
	Proportion with creatinine results in the previous year	49.507	(−18.277; 117.291)

eGFR	Structure points	0.678	(−0.479; 1.835)
	Process points	−2.212	(−7.321; 2.896)
	Proportion with HbA1c results in the previous year	48.127	(−7.223; 103.477)
	Proportion with creatinine results in the previous year	−62.586	(−114.121; −11.051)

SBP	Structure points	0.021	(−0.815; 0.857)
	Process points	0.343	(−3.557; 4.244)
	Proportion with HbA1c results in the previous year	−21.008	(−61.717; 19.701)
	Proportion with creatinine results in the previous year	10.109	(−27.227; 47.446)

BMI	Structure points	0.100	(−0.079; 0.279)
	Process points	−0.217	(−1.025; 0.591)
	Proportion with HbA1c results in the previous year	−1.851	(−10.453; 6.751)
	Proportion with creatinine results in the previous year	−1.450	(−9.432; 6.532)

*β*: beta coefficient; CI: confidence interval; HbA1c: glycated hemoglobin; LDL: LDL cholesterol; eGFR: estimated glomerular filtration rate (MDRD); SBP: systolic blood pressure; BMI: body mass index.
